# Granular cell tumor of the neck treated using a local flap: A case report

**DOI:** 10.1097/MD.0000000000032641

**Published:** 2023-02-22

**Authors:** Chan Mi Lee, Yong Tae Hong

**Affiliations:** a Department of Otolaryngology-HNS, Seoul National University Hospital, Seoul, Korea; b Department of Otolaryngology-HNS, Research Institute for Clinical Medicine of Jeonbuk National University- Biomedical Research Institute of Jeonbuk National University Hospital, Jeonbuk, Korea.

**Keywords:** granular cell tumor, granulocytoma, neck mass

## Abstract

**Rationale:**

Granular cell tumors are rare soft tissue neoplasms derived from nerve that can arise in the oral cavity, skin, or gastrointestinal tract. Various hypotheses have proposed that granular cell tumors originate from the nervous system, skeletal muscle, and Schwann cells.

**Patient concerns:**

A 47-year-old male patient presented with a 5 cm cervical mass.

**Diagnoses:**

Computed tomography showed a 4 cm-sized homogeneous enhancing mass infiltrating the sternocleidomastoid muscle and even the surrounding skin.

**Interventions:**

Extensive surgical resection of the tumor including the skin was performed. A submental transposition local flap was used for the wide skin defect.

**Outcomes:**

Histologic finding showed polygonal granular cells with rich eosinophilic coarse granular cytoplasm without interstitial tissue without mitosis or necrosis. Immunohistochemically, the tumor cells were positive for S100 and CD68, which is consistent with classic granular cell tumors.

**Lessons:**

In microscopic observations, granular cell tumors do not have a defined boundary with surrounding tissues, and they display an infiltrating pattern that can expand to adjacent tissue. As a result, the tumor should be removed with a sufficient margin, including the normal tissues surrounding it. The authors experienced granular cell tumor in the muscle layer of the head and neck. It could be treated without recurrence through extensive surgical resection and local flap.

## 1. Introduction

Granular cell tumors are rare soft tissue neoplasms derived from nerve and can arises at oral cavity, skin, or gastrointestinal tract.^[1]^ Various hypotheses have proposed that the origin of the granular cell tumors was nervous system or skeletal muscle or Schwann cell.^[2]^ The characteristic pathologic finding is polygonal granular cells with rich eosinophilic coarse granular cytoplasm.^[3]^

We experienced a granular cell tumor, 5 cm-sized, fixed cervical mass. Here, we report a 47 year-old male patient presented with a right cervical granular cell tumor, and discuss its characteristics and treatment.

## 2. Case report

**Figure 1.**
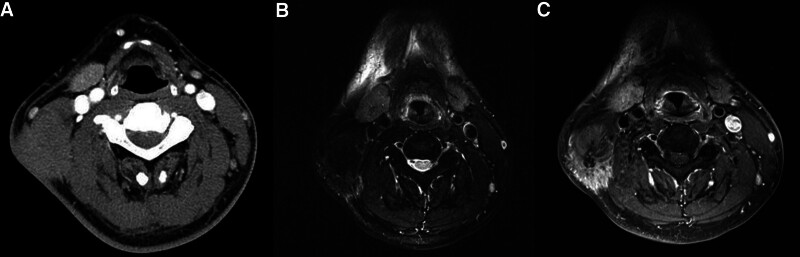
(A) CT, axial homogeneous enhancing mass infiltrating the sternocleidomastoid muscle and skin (B) MRI T2WI shows a mass infiltrating the SCM muscle with low signal intensity (C) MRI TIWI images shows a mass with similar signal intensity to muscle and had heterogeneous enhancement. CT = computed tomography, MRI = magnetic resonance imaging, SCM = sternocleidomastoid.

**Figure 2.**
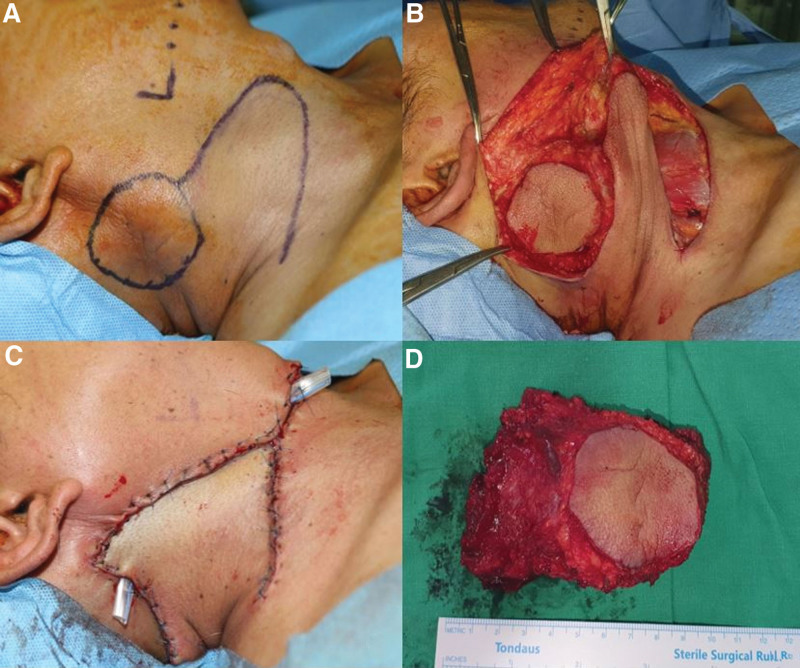
Submental transposition flap was designed on the right neck and chin, and the skin defect was reinforced by rotation flap.

A 47-year-old male patient presented with a right cervical mass that occurred several years ago. On physical examination, a 5 cm-sized, fixed, non-tender mass was palpated in the right cervical level II area. And he had no family history or medical history. Computed tomography showed a 4 cm-sized homogeneous enhancing mass infiltrating the sternocleidomastoid muscle and even the surrounding skin (Fig. 1). In magnetic resonance imaging, a 4.8 cm sized mass infiltrating the sternocleidomastoid muscle showed low signal intensity at T2WI, similar signal intensity to muscle at TIWI and had heterogeneous enhancement. The mass was extended to the subcutaneous layer, some of the boundaries were smooth margin, but some were infiltrative margin, suspicious of soft tissue origin sarcoma. (Fig.1). In the core needle biopsy, it was estimated to be a granular cell tumor. modified radical neck dissection was performed and submental transposition local flap was used for skin defect. Internal jugular vein, carotid artery and spinal accessory nerve was preserved and the submental transposition flap was designed on the right neck and chin, and the skin defect was reinforced by rotation flap (Fig.2).

**Figure 3.**
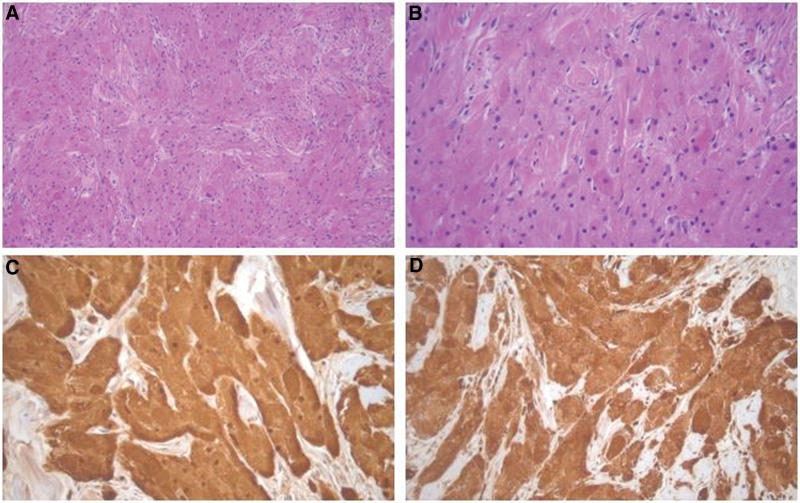
Histologic findings of granular cell tumor. (A) The tumor is composed of sheets and nests of large polygonal cells. (B) The tumor cells are polygonal and had characteristic eosinophilic and granular cytoplasm. Immunohistochemically, the tumor cells are positive for (C) S100 and (D) CD68. Original magnifications: (A) x200. (B–D) x400.

There were no diagnostic challenges. Histologic finding showed polygonal granular cells with rich eosinophilic coarse granular cytoplasm without interstitial tissue without mitosis or necrosis (Fig. 3). Immunohistochemically, the tumor cells are positive for S100 (Fig. 3C) and CD68 (Fig. 3D) consistent with classic granular cell tumor. There were no complications such as facial paralysis after surgery and no recurrence during the 6-month follow-up period. Informed consent was obtained from the patient for publication of this case report.

## 3. Discussion

In 1926, Abrikossoff named granulocytoma “Myoblastic myoma,” with a focus on differentiation from skeletal muscles, while in 1935,^[4]^ Feyrter proposed the origin was from nerve,^[2]^ and in 1962, using an electron microscope, Fisher and Wechsler suggested the Schwann cell origin hypothesis.^[5]^ Because the cause is still unknown, the term “granular cell tumor” has been used to describe it.^[3]^ The lesion can appear anywhere, however it is most common in the head and neck.^[6]^ The tongue is the most common caused lesion, with 23 to 28% of all granular cell tumors occurring there.^[7]^

A painless nodule in the dermis or subcutaneous layer, or rarely in mucous membranes, skeletal or smooth muscles, is the most prevalent symptom. Malignancy is typically greater, with an average diameter of 4.0 cm observed by Fanburg-Smith.^[8]^ In microscopic observations, granular cell tumors do not have a defined boundary with surrounding tissues, and they display an infiltrating pattern that can expand to adjacent tissue. As a result, the tumor should be removed with a sufficient margin, including the normal tissues surrounding it.^[4]^ Although there are no histological diagnostic criteria for malignant granular cell tumors, polymorphism, enhanced mitosis, necrosis, and nuclear/cytoplasm rates are the most important features, which are linked to high levels of p53 and Ki67.^[8]^ However, some cases of metastasis are found to have benign microscopic results. Gamboa described some cases of malignant granular cell tumors that were histologically benign but clinically malignant in 1995.^[9]^ If the tumor is >4 cm and has grown rapidly or recurred locally in recent years, malignant granular cell tumor should be suspected.^[10]^ Due to significant tumor resistance, wide surgical excision is the primary treatment for benign and malignant tumors, and radiation therapy or chemotherapy are not advised.^[1]^ Recurrence of a benign granular cell tumor is uncommon, however it is frequently related with insufficient resection.^[11]^

The authors experienced granular cell tumor in the muscle layer of the head and neck. It could be treated without recurrence through extensive surgical resection. Pathological examination showed no suspected malignant findings, and clinically surrounding tissue infiltration was not clear. Therefore, it was not suitable to malignancy, but attention should be paid to complete resection with sufficient resection margin in the early stages.

## 4. Conclusion

The authors experienced granular cell tumor in the muscle layer of the head and neck. It could be treated without recurrence through extensive surgical resection. Submental transposition local flap was used for wide skin defect. It was designed on the right neck and chin, and the skin defect was reinforced by rotation flap. During the 6-month follow-up period, there were no complications such as facial paralysis after surgery and no recurrence.

## Acknowledgments

We would like to acknowledge the Research Institute for Clinical Medicine of Jeonbuk National University- Biomedical Research Institute of Jeonbuk National University Hospital for supporting this study.

## Author contributions

**Conceptualization:** Yong Tae Hong.

**Writing – original draft:** Chan Mi Lee.

**Writing – review & editing:** Yong Tae Hong, Chan Mi Lee.
